# The South African Rugby Injury and Illness Surveillance and Prevention Project (SARIISPP): *SARU Youth Week Injury Surveillance Report* 2019

**DOI:** 10.17159/2078-516X/2020/v32i1a9257

**Published:** 2020-01-01

**Figure:**
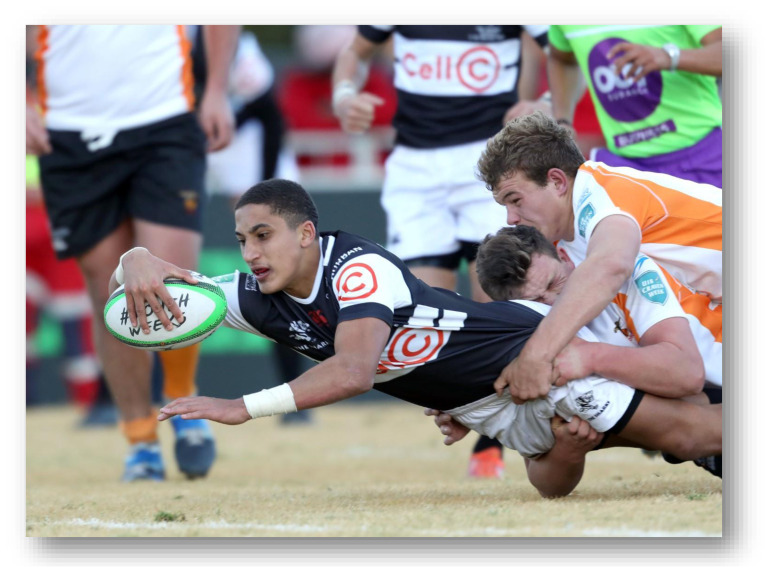


## Executive Summary

As part of the South African Rugby Injury and Illness Surveillance and Prevention Project (SARIISPP), SA Rugby investigates rugby injuries at the annual SARU Youth Week tournaments. The BokSmart National Rugby Safety Programme has been collecting robust standardised data at these tournaments since 2011. Analysing these data annually shows injury patterns over time between tournaments and collectively, for all the SARU Youth Weeks. Also, the analysis compares the profiles of injured players at each individual tournament.

When investigating these patterns, areas of concern are identified, changes in the game, tournament structure or medical support services are considered or refuted against the evidence, and injury specific interventions can be created and implemented, where the evidence indicates such a need.

Each medical facility at the SARU Youth Week tournaments has a designated researcher onsite, who together with the tournament medical doctor, records the tournament injury data daily. If additional information was required, the researcher followed up regularly with the injured player after the tournament to ensure that all necessary injury data were captured.

This 2019 SARU Youth Week report focuses on the boy’s tournaments, comprising of the u13 Craven Week (CWu13), u16 Grant Khomo Week (GKu16), u18 Academy Week (AWu18) and u18 Craven Week (CWu18). The tournaments consisted of 84 teams and 130 matches. Additionally, comparisons are made between SARU Youth Week tournaments and over time between 2011 and 2019.

In 2019, the GKu16 recorded the highest Time-Loss injury incidence at 26 (16–37) [mean (95% confidence intervals)] injuries per 1000 player hours and the CWu13 the lowest of all the SARU Youth Weeks at 5 (0–10) injuries per 1000 player hours. The CWu13 was the lowest recorded injury incidence to date at the SARU Youth Weeks since SARIISPP was introduced in 2011. The collective tournament average was measured at 18 (14–22) Time-Loss injuries per 1000 player hours or roughly 1 Time-Loss injury for every 2 matches played. When combining the injury incidence data collected over the past nine years, CWu18 has the lowest Time-Loss injury incidence, and GKu16 the highest.

In 2019, the Tackle and the Ball Carry, followed by the Ruck, were the most frequent injury-causing events in that order. Tackling front-on (*regulation*) for the Tackler, being tackled front-on (*regulation*) for the Ball Carrier and being kicked during the Ruck were respectively the most frequent injury mechanisms involved in these three phases of play.

The most common injury type was Joint/Ligament injuries, where AWu18 and CWu18 recorded the highest incidence of these. In the 2019 tournaments the most common injury location was the Lower Body, with most of these injuries occurring in the CWu18. Props and Locks had the highest injury incidence across all tournaments and Scrumhalves, Flyhalves and Fullbacks had the lowest injury incidence.

As expected, the injury incidence of *‘New’* injuries was higher than subsequent ‘*Recurrent’* injuries; the majority of ‘*New*’ injuries being injuries to the muscle, while most *‘Recurrent’* injuries were ligament injuries.

The majority of injured players (88%) started the match.

Only 14 concussions occurred across all the tournaments, which is a record low. The GKu16 had the highest concussion incidence of all the tournaments. The Tackler and the Ball Carrier roles were the events causing the most concussions, followed by the Ruck.

## Definitions

All definitions are originally based on the 2007 consensus statement for injury reporting in rugby union (1) and have since been realigned with the latest International Olympic Committee (IOC) consensus statement for methods of recording and reporting epidemiological data on injury and illness in sport (2–3).

### MEDICAL ATTENTION INJURY

Injury, according to the International Olympic Committee Consensus Statement of 2020, can be defined as *“tissue damage or other derangement of normal physical function due to participation in sports, resulting from rapid or repetitive transfer of kinetic energy”* (2). All injuries that were seen by the Tournament Medical Doctors were classified as Medical Attention injuries, which are defined by the 2007 statement as an “*injury that results in a player receiving medical attention”* (1), and by the more recent IOC statement as *“a health problem that results in an athlete receiving medical attention”* (2–3).

### TIME-LOSS INJURY

Medical Attention injuries were further categorised as Time-Loss injuries, where appropriate, and defined by the 2007 statement as, “*an injury that results in a player being unable to take a full part in future rugby training or match play*” (1). The IOC definition is, *“a health problem that results in a player being unable to complete the current or future training session or competition”* (2). In this report it is specific to injuries (3).

### INJURY RATE

For this 2019 SARU Youth Week report, an injury rate is the number of injuries expressed per 1000 player exposure hours. This normalised version of the number of injuries has been used in previous reports and enables comparison between current tournaments, previous tournaments and to other published scientific literature. Moreover, the injury rate is expressed as a mean with 95% confidence intervals. A 95% confidence interval around a mean value indicates that we can be 95% certain that the value is bounded by the two intervals. In this report, we present the 95% confidence intervals assuming normal distribution of the data and use the approach of examining the overlap of the confidence intervals to determine whether the injury incidences are significantly different. If the range of confidence interval values of two comparisons do not overlap, there is a strong (95%) chance that their injury rates are different from each other. This method is conservative and is less likely to produce false positive results (3–4).

### NEW, SUBSEQUENT AND RECURRENT INJURIES

In the 2019 SARU Youth Week report, a ‘*New Injury’* was defined as when a player sustained his first injury in the tournament. Any injury that the *same* player sustained after this initial injury was defined as a *‘Subsequent Injury’*.

According to the more recent IOC statement, any subsequent injury to the same site and of the same type is referred to as a ‘*Recurrence’* if the index injury was fully recovered before reinjury, and as an *‘Exacerbation’* if the index injury was not yet fully recovered (2).

To provide more detail on the subsequent injuries for practitioners, we have further categorized the subsequent injuries in this report into one of four groups based on the OSICS classification diagnosis (5):

- Different site - Different type
- Different site - Same type
- Same site - Different type
- Same site - Same type

According to the 2007 Consensus Statement for rugby, and due to the timing and nature of the tournament, any subsequent injury classified as ‘Same site - Same type’ was classified as a *‘Recurrent injury’* (1,3).

**Figure:**
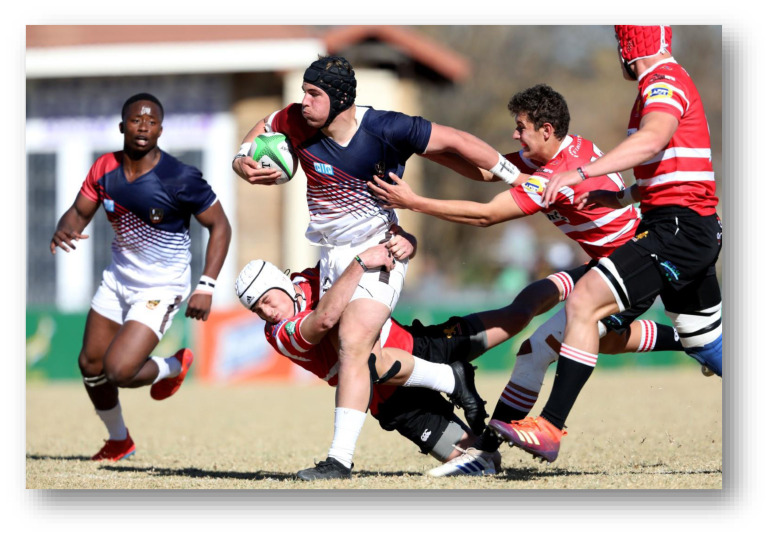


## Key Findings

### Injury Incidence

**Figure 1:**
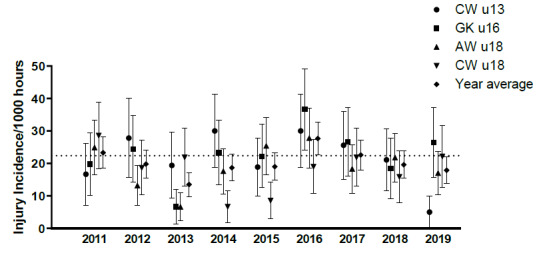
Injury incidence/1000 player hours and 95% confidence intervals of Time-Loss injuries for the SARU Youth Week Tournaments from 2011 – 2019. The dotted line reflects the average incidence for all tournaments over all the included years.

Eighty-four teams competed in the 2019 SARU Youth Week tournaments (CWu13 = 18 teams, GKu16 = 20 teams, AWu18 = 28 teams, CWu18 = 18 teams). A total of 183 Medical Attention injuries were recorded during all the tournaments in 2019; forty percent of these (n = 73) were Time-Loss injuries. The combined tournaments’ injury incidence and 95% confidence intervals for all Medical Attention injuries was 45 (38 to 51) injuries/1000 player hours, and for Time-Loss injuries was 18 (14 to 22) injuries/1000 player hours. The incidence of Medical Attention injuries in the AWu18 and the incidence of Time-Loss injuries in the CWu13 respectively, were significantly lower than any of the other SARU Youth Week tournaments in 2019 (Table 1). The number of Time-Loss injuries in the CWu13 tournament was the lowest number recorded in the CWu13 tournaments since 2011. The numbers of Medical Attention and Time-Loss injuries per match and per hour of match play across the tournaments are represented in Table 2. Figure 1 shows the pattern of Injury incidence/1000 player hours and 95% confidence intervals of Time-Loss injuries for each tournament across the years (2011 to 2019) (Figure 1).

**Figure 2:**
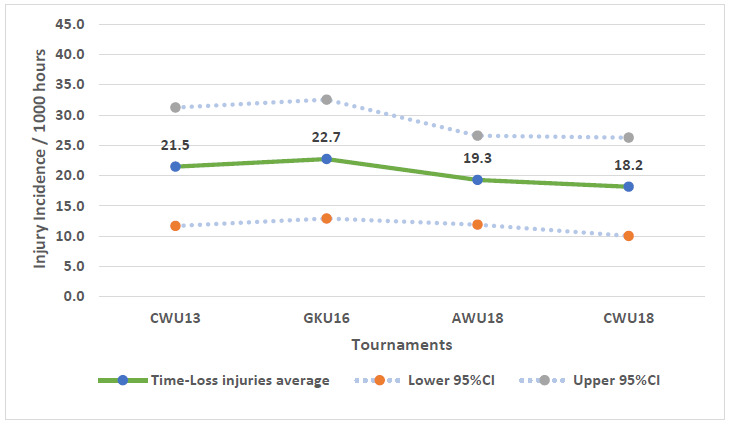
Injury incidence/1000 player hours and 95% confidence intervals (dotted blue lines) at the SARU Youth Week tournaments from 2011 – 2019.

Further analysis was completed on Time-Loss injuries only. Combined data from 2011 to 2019 shows a slight increase in injury incidence from CWu13 to GKu16. This then decreases from GKu16 to AWu18 and continues to decrease with the injury incidence lowest at the CWu18 tournaments over the 9 years studied (Figure 2). Overall, the statistics suggest there is little difference in injury incidence rates between the tournaments.

### Injury Incidence Trends

#### U13 Craven Week (CWu13)

**Figure 3:**
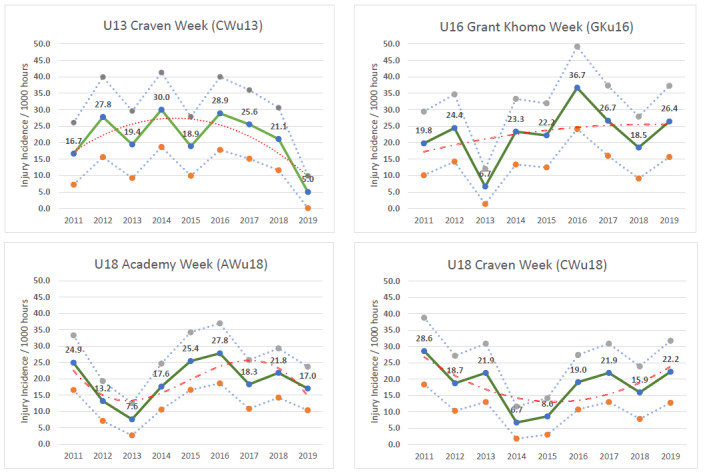
Time-Loss Injury incidence for each SARU Youth Week tournament, per year, from 2011 – 2019, including the upper and lower 95% Confidence Intervals (95%CI). The dashed orange line represents the polynomial trend.

There was an alternating increase-decrease pattern in injury incidence in the CWu13 between 2011 and 2017. In 2019, however, a decrease in injury incidence was observed for the third consecutive year. In 2019, the injury incidence was the lowest recorded in the past 9 years (Figure 3). In Figure 3, the polynomial trendline accounts for 60% of the variance in injury incidence per 1000 player hours (R^2^ = 0.60), which highlights the downward trend from 2016 to 2019.

#### U16 Grant Khomo Week (GKu16)

There was a sizable decrease in injury incidence between 2016 and 2018. However, in 2019, the GKu16 injury incidence rebounded again to its third highest rate recorded over the past 9 years (Figure 3) and this was similar to the 2017 data. In Figure 3, the polynomial trendline accounts for only 10% of the variance in injury incidence per 1000 player hours (R^2^ = 0.10). The trendline increases and then levels out from 2018 to 2019.

#### U18 Academy Week (AWu18)

Following a sizable decrease in injury incidence at the AWu18 between 2016 and 2017, it remains lower but has been fluctuating with a slight increase noted in 2018 followed by a decrease in 2019 (Figure 3). In Figure 3, the polynomial trendline accounts for 50% of the variance in injury incidence per 1000 player hours (R^2^ = 0.50). The trendline fluctuates throughout the years, however, has a downward trend from 2017 to 2019.

#### U18 Craven Week (CWu18)

Injury incidence gradually increased each year between 2014 and 2017 and then decreased in 2018. In 2019, injury incidence rebounded again to the second highest incidence rate recorded over the past 9 years (Figure 3), albeit quite similar to 2017. In Figure 3, the polynomial trendline accounts for 50% of the variance in injury incidence per 1000 player hours (R^2^ = 0.50). The trendline shows an increase in injury incidence since 2015.

**Figure:**
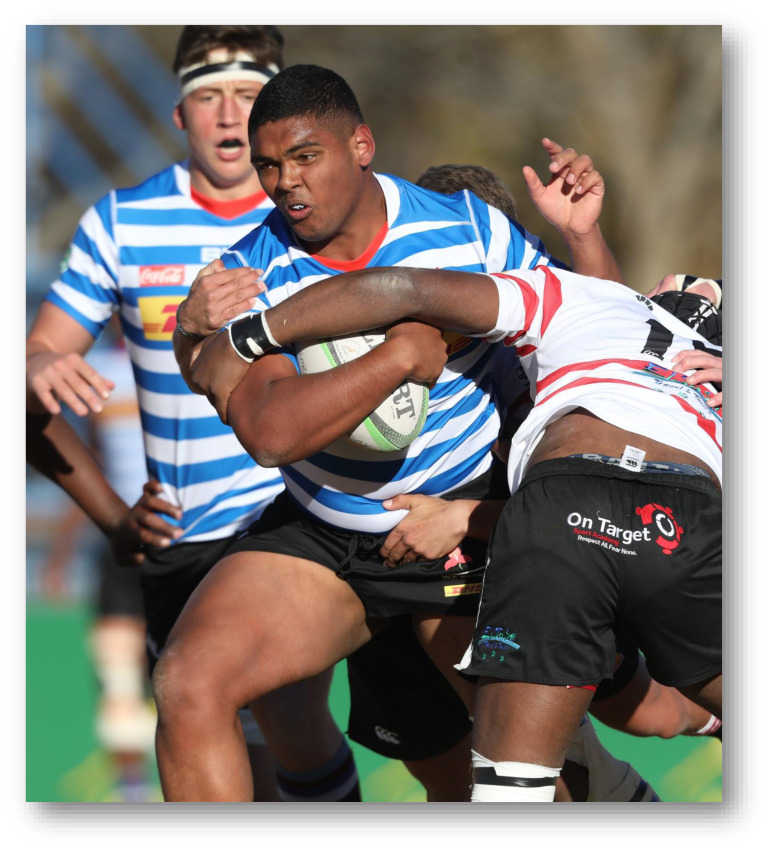


**Figure:**
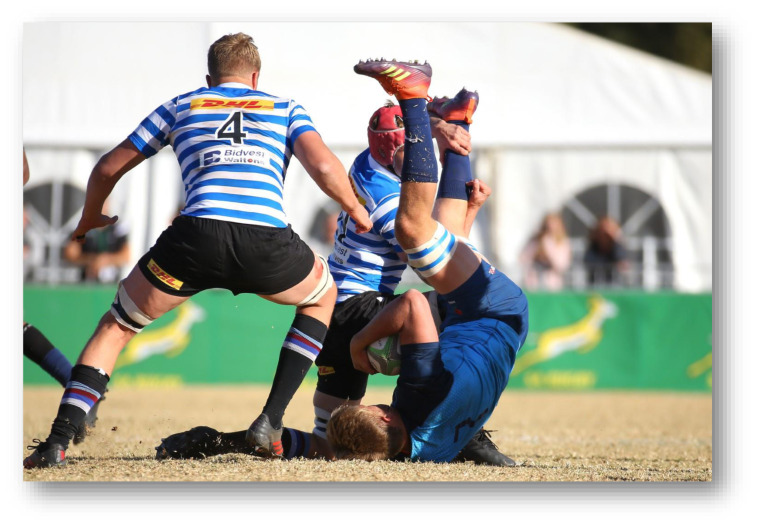


### Injury Event

When all tournaments in 2019 were combined, the Tackler and Ball Carrier roles (both at 29%) were associated with the most injuries, followed by the Ruck (25%). Tacklers and Ball Carriers had 5 (3 to 7) injuries/1000 player hours, while Rucks had 4 (2 to 6) injuries/1000 player hours.

Injury incidence to the Tackler was highest in the CWu18 tournament. Ball Carrier injury incidence was highest in the GKu16 (Table 3). Ruck injuries were more present in the GKu16 and AWu18 tournaments.

**Figure 4:**
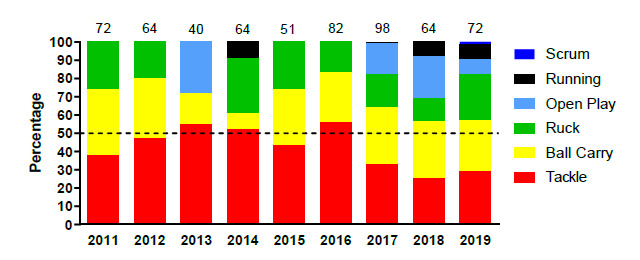
Most common injury causing events in the SARU Youth Week tournaments from 2011 – 2019. (The number above each bar represents the total number of injuries for that year). Missing 2019 data = 1 case.

Figure 4 displays the proportion of injuries resulting from the different injury causing events between 2011 and 2019. There was a marginal increase in the proportion of injuries sustained by the Tackler in 2019 compared to the previous year but to a level similar to that of 2017. It nonetheless remained lower than the proportion of injuries sustained by the tackler between 2011 and 2016.

The Ruck phase of play however doubled in percentage from 2018 to 2019. A decrease in the proportion (15%) of open play injuries was also noted in 2019 (Figure 4).

**Figure 5:**
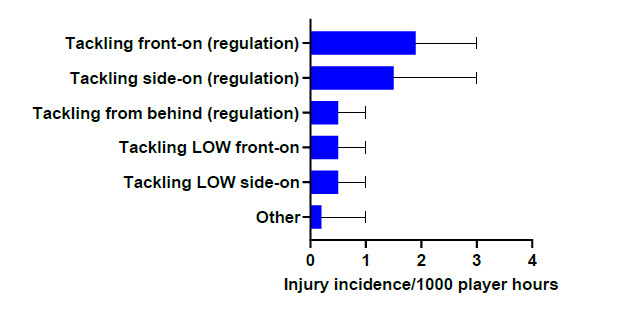
Injury incidence and 95% confidence intervals/1000 player hours of Tackler-related injury mechanisms at the 2019 SARU Youth Week Tournaments.

In 2019, Tackling front-on (regulation) accounted for the highest proportion of injuries to the Tackler (38%) with 1.9 (1 to 3) injuries/1000 player hours. Tackling side-on (regulation) accounted for 29% of injuries sustained by the Tackler with 1.5 (0 to 3) injuries/1000 player hours (Figure 5).

**Figure 6:**
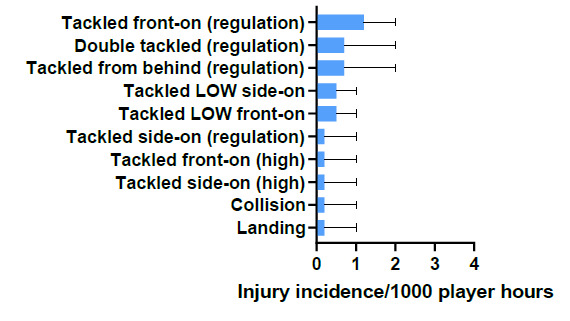
Injury incidence and 95% confidence intervals/1000 player hours of Ball Carrier-related injury mechanisms at the 2019 SARU Youth Week Tournaments.

Tackled front-on (regulation) accounted for the highest proportion of injuries to the Ball Carrier (25%), with 1.2 (0 to 2) injuries/1000 player hours (Figure 6).

**Figure 7:**
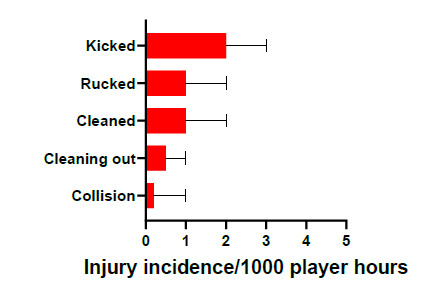
Injury incidence and 95% confidence intervals/1000 player hours of Ruck-related injury mechanisms at the 2019 SARU Youth Week Tournaments.

In 2019, being kicked accounted for the highest proportion of injuries in the Ruck (33%) with 2 (0 to 3) injuries/1000 player hours. Being *cleaned out* accounted for 22% of injuries sustained in the Ruck, in comparison to the act of *cleaning out* (11%) (Figure 7).

### Injury Type

In the 2019 SARU Youth Week tournaments the most common injury type was Joint/Ligament injuries (Table 4). There were significantly more Joint/Ligament injuries than Muscle/Tendon injuries at the AWu18 and in the combined data across all tournaments. Other than that, there were no significant differences for any of the within tournament injury type comparisons.

The AWu18 and CWu18 tournaments had the highest incidence of Joint/Ligament injuries.

**Figure 8:**
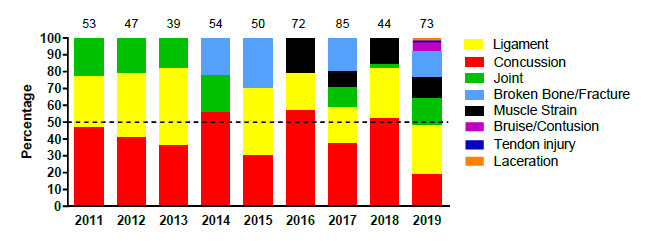
Most common injury types in the SARU Youth Week tournaments from 2011 – 2019. (The number above each bar represents the total number of injuries for that year).

Figure 8 displays the most common injury types in proportionate contribution per year from 2011 to 2019. Concussions contributed much less in 2019 than in 2018, and joint and bone-related injuries substantially more. Both Joint injuries and Broken Bone/Fractures increased noticeably in 2019.

**Figure:**
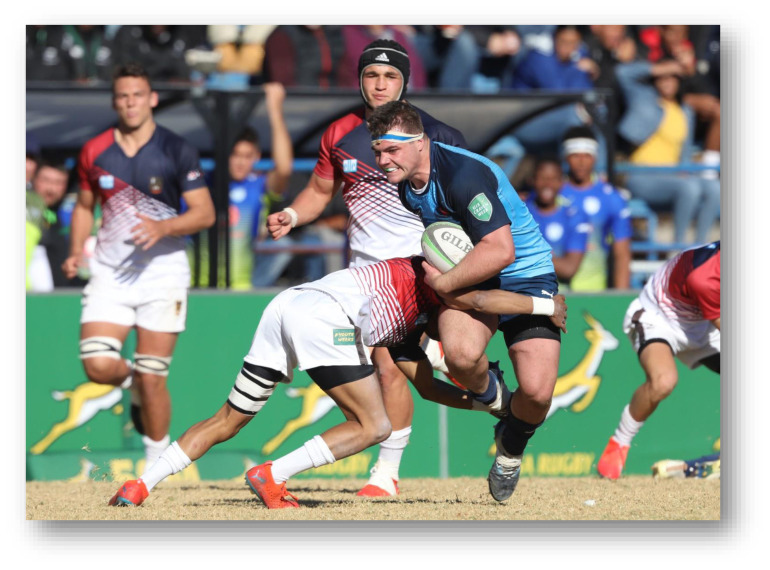


### Body Location

All the injuries from the 2019 tournaments were grouped according to the four main body location groups (*Head & Neck; Trunk; Upper Body; Lower Body*). In the 2019 tournaments the most common injured body location was the Lower Body (38%), with 43% of these occurring at the CWu18 tournament. Lower Body injuries recorded an injury incidence of 7 (4–9) injuries/1000 player hours (Table 5). The CWu18 had the highest Lower Body injury incidence at 13 (6 to 20) injuries/1000 player hours, but this was not significantly different from the other SARU Youth Weeks. Trunk injuries were significantly lower than the other body locations.

Table 6 presents the 2019 SARU Youth Week Tissue and Pathology injury data in the format recommended by the most recent IOC consensus statement (2).

### New Vs Recurrent

The injury incidence of *‘New’* injuries in 2019 was 11 (8 to 15) injuries/1000 player hours; a lower injury incidence than in 2018. The subsequent ‘*Recurrent’* injuries were 6 (4 to 9) injuries/1000 player hours, which was slightly higher than in 2018.

Most of the *‘New’* injuries were to the muscle (78%), while most *‘Recurrent’* injuries were ligament injuries (43%).

**Figure 9:**
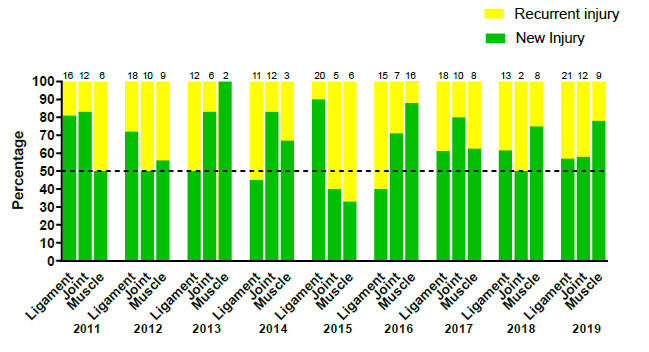
Proportion of New and Recurrent ligament, joint and muscle injuries in the SARU Youth Week tournaments from 2011 – 2019. (The number above each bar represents the total number of injuries for that year).

Figure 9 illustrates the proportion of *‘New’* and *‘Recurrent’* ligament, joint and muscle injuries across the years (2011–2019). There was a slight increase in the proportion of *‘Recurrent’* ligament injuries from 2018 (39%) to 2019 (43%), while there was a decrease in the proportion of *‘Recurrent’* joint injuries from 2018 (50%) to 2019 (42%) and *‘Recurrent’* muscle injuries from 2018 (25%) to 2019 (22%).

There was a corresponding decrease in the proportion of *‘New’* ligament injuries from 62% in 2018, to 57%, in 2019. There was also a corresponding increase in the proportion of *‘New’* joint injuries and muscle injuries from 50% and 75% in 2018, to 58% and 78% in 2019.

### Game Quarter

**Figure 10:**
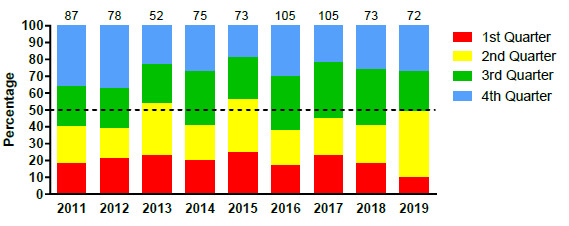
Proportion of injuries occurring in each game quarter in the SARU Youth Week tournaments from 2011 – 2019. (The number above each bar represents the total number of injuries for that year). Missing data in 2019 = 1 case.

Injuries for the 2019 tournaments occurred mostly in the 2^nd^ quarter (39%); 7 (4 to 9) injuries/1000 player hours. The increase from 2018 to 2019 in 2^nd^ quarter proportionate contributions to injuries is seemingly large, however, there was no significant change in the distribution of injuries between quarters across the years (Figure 10).

### Match status

**Figure 11:**
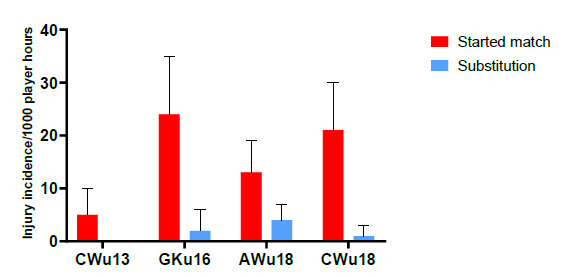
Injury incidence and 95% confidence intervals/1000 exposure hours of players who started the match and those who came on as substitutes, in the 2019 SARU Youth Week Tournaments.

In 2019, there were significantly more injuries to players who started the match (88%) compared to players who joined the match as substitutions (12%) (Figure 11). CWu13 had a significantly lower injury incidence/1000 player hours to their starting line-ups in comparison to the GKu16 and CWu18 tournaments at 5 (0 to 10) injuries/1000 player hours and had no substitution injuries. There were no other significant differences in injury rates between tournaments or in players who came on as substitutes (Table 7).

### Player positions

**Figure 12:**
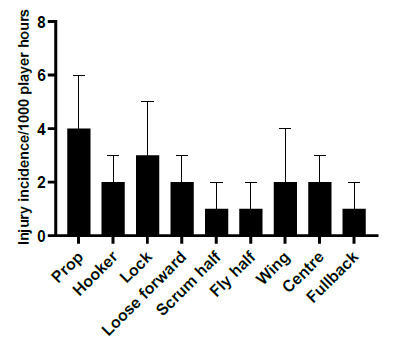
Injury incidence and 95% confidence intervals/1000 player hours per position in the SARU Youth Week Tournaments 2019.

**Figure 13:**
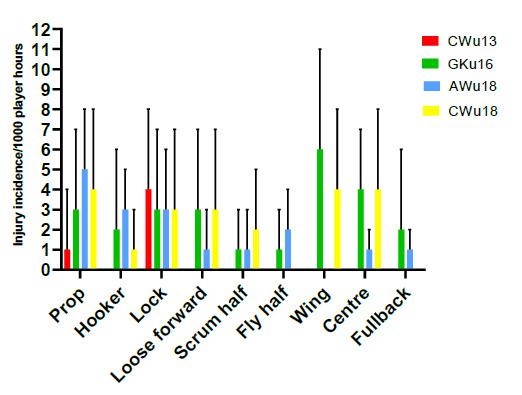
Injury incidence and 95% confidence intervals/1000 player hours per position across the various SARU Youth Week Tournaments in 2019.

In 2019, players in the prop and lock positions had the highest injury incidence rates across all tournaments. Props had an injury incidence of 4 (2 to 6) injuries/1000 player hours and locks 3 (2 to 5) injuries/1000 player hours. Scrumhalves, flyhalves and fullbacks shared the lowest injury incidence at 1 (0 to 2) injury/1000 player hours (Figure 12). Figure 13 illustrates the injury incidence of player positions across the various tournaments. Props and locks were the only injured players in the CWu13 tournament (Figure 13).

**Figure:**
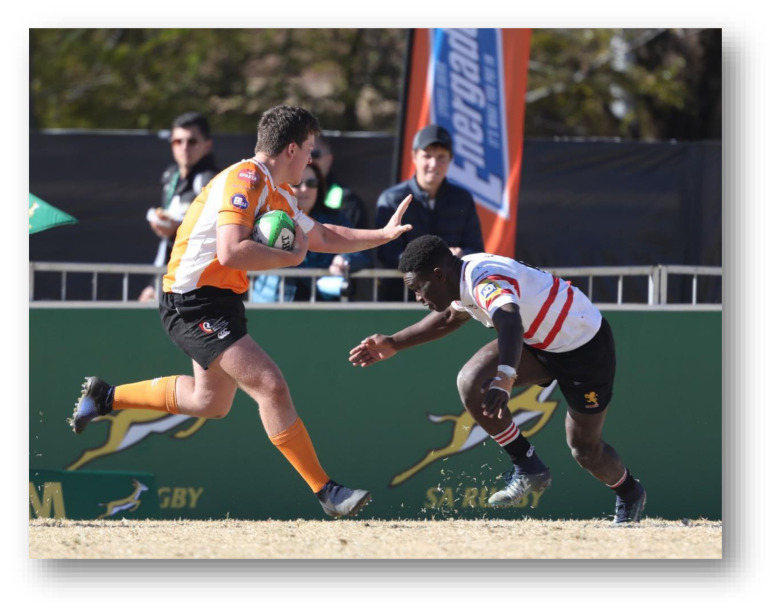


### Concussion

There was a total of 14 concussions recorded over all the tournaments played in 2019. This was the lowest number of concussions recorded to date and converts to an incidence rate of 3 (2 to 5) concussions/1000 player hours and roughly one concussion for every 9 matches played.

The GKu16 was the tournament with the highest concussion incidence rate of 7 (1 to 12) concussions/1000 player hours (Table 8). These data converted to 5 matches/concussion event. The CWu13 had the lowest tournament concussion rate with one concussion occurring within 32 matches played (Table 8).

The tournament concussion rates were not significantly different from each other. This however should be interpreted with caution because of the low statistical power as a result of the small sample size.

**Figure 14:**
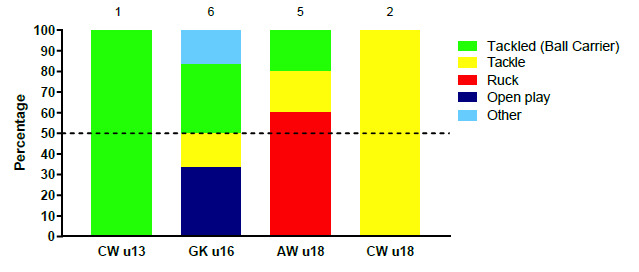
Proportion of concussions caused by the different injury events at the 2019 SARU Youth Week Tournaments (n = 14 concussions).

**Figure 15:**
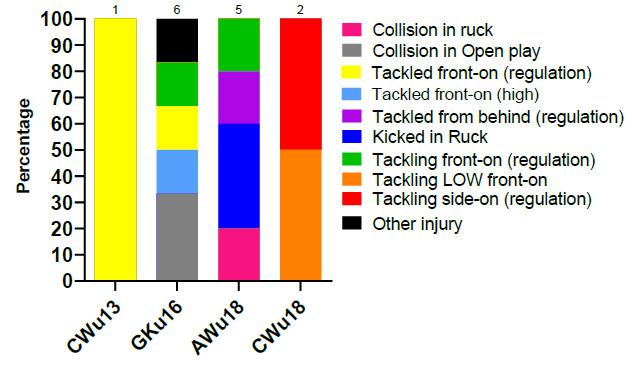
Proportion of concussions caused by the different injury mechanisms at the 2019 SARU Youth Week Tournaments (The number above each bar represents the total number of concussions for that tournament).

In 2019, Tackling and being Tackled (both at 29%, n = 4), and competing in the Ruck (21%, n = 3), were the three events that contributed to the most concussions (Figure 14). Figure 15 displays the proportion of concussions caused by the different injury mechanisms across the tournaments in 2019. Although the numbers are too low to make any firm conclusions, the front-on tackle event including the roles of both Tackler and Ball Carrier, was the most prominent concussion causing event, contributing to 6 of the 14 cases in 2019.

**Figure 16:**
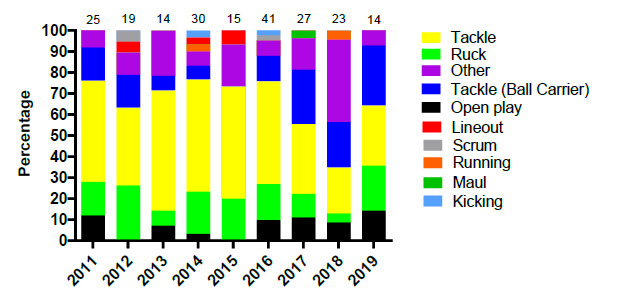
Proportion of concussions caused by the different injury events from 2011 to 2019 SARU Youth Week Tournaments. (The number above each bar represents the total number of concussions for that year).

In Figure 16, not only has the number of concussions decreased since 2011 but the amount of injury mechanisms causing concussions has decreased.

**Figure 17:**
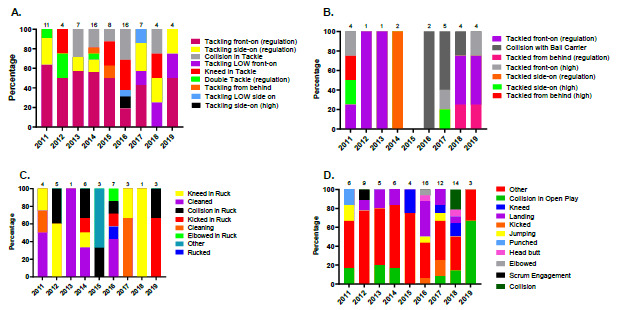
Proportionate breakdown of concussions caused by the various injury mechanisms at the 2011 to 2019 SARU Youth Week Tournaments. (The number above each bar represents the total number of concussions for that year). A. Tackler-related concussion mechanisms B. Ball Carrier-related concussion mechanisms C. Ruck-related concussion mechanisms D. Remaining concussion mechanisms.

Figure 17 displays the proportion of injuries resulting from the different injury mechanisms causing concussions over the 9 years studied.

Between 2011 and 2019, 44% of Tackler-related concussions (Figure 17A) were caused by tackling front-on (regulation), 30% of Ball Carrier-related concussions (Figure 17B) were caused by being tackled front-on (regulation) and 24% of Ruck-related concussions were caused by being cleaned out (Figure 17C).

**Figure 18:**
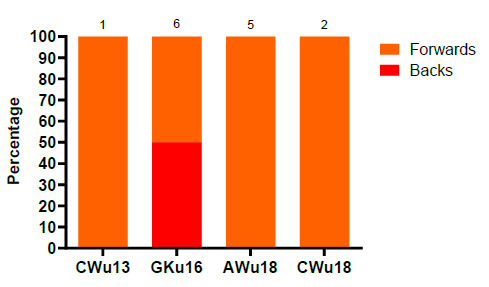
Proportionate breakdown of concussions for forwards and backs at the 2019 SARU Youth Week Tournaments (The number above each bar represents the total number of concussions for that tournament).

The majority of the concussions in 2019 occurred to forwards (79%). Only 21% of concussions were experienced by backs, and all of these were in GKu16 (Figure 18). In 2019, the most prominent position for concussions were locks (29%) followed by loose-forwards (21%). Scrumhalves, wings and fullbacks each accounted for 7% of concussions.

**Figure:**
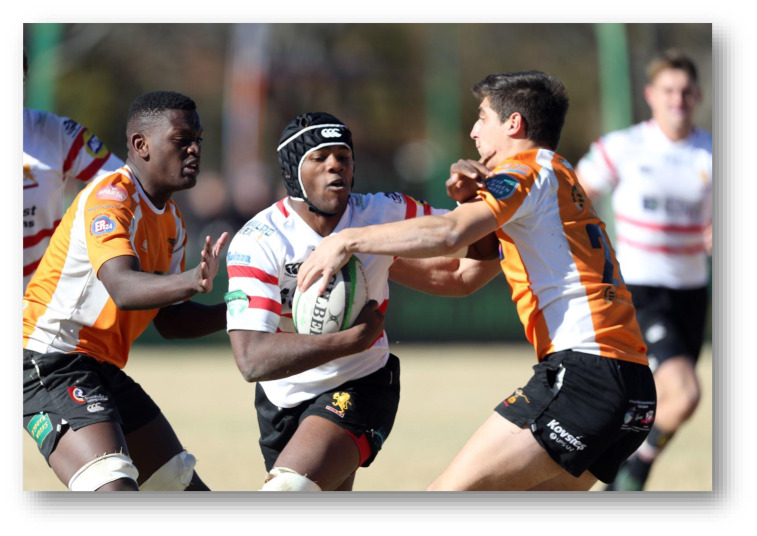


**Figure 19:**
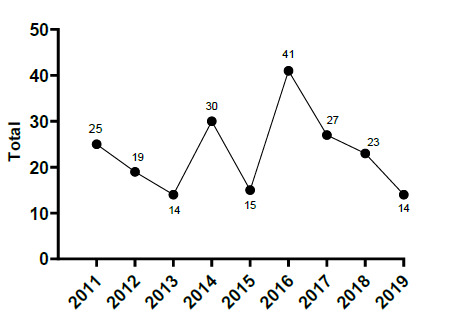
Total number of concussions per year at the SARU Youth Week Tournaments from 2011 – 2019. (The number above each data point represents the total number of concussions for that year).

**Figure 20:**
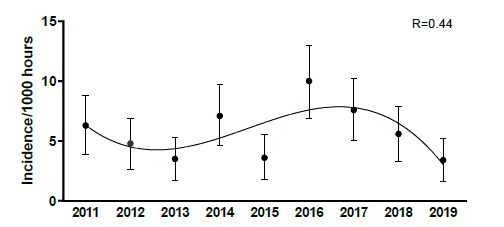
Concussion incidence rates and 95% confidence intervals/1000 player hours per year at the SARU Youth Week Tournaments from 2011 – 2019.

When looking at the total number and rate of concussions from 2011 to 2019, the pattern for both total number (Figure 19) and rate (Figure 20) suggests an initial increase in concussions up until 2016 and then a gradual decrease continuing into 2019. This pattern can be attributed to the interaction of several factors including greater awareness, evolving medical protocols and training techniques, and improved sensitivity in recognizing, managing and preventing concussions (Figures 19 and 20). Due to the heightened awareness and strict concussion protocols in place at that time, there is no specific reason or explanation for the outlier value in 2015 and to try and explain this would purely be speculative.

**Figure 21:**
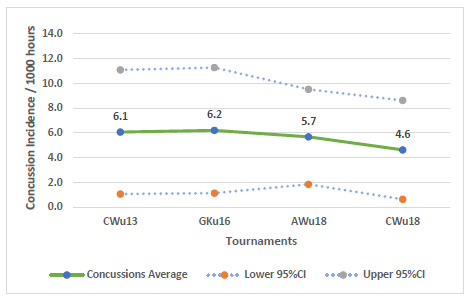
Concussion incidence rates and 95% confidence intervals/1000 player hours per SARU Youth Week tournament from 2011 – 2019.

There is a tendency for the combined concussion injury incidence (2011 – 2019) to decrease as the age and level of the players increased (Figure 21), but these were not significantly different.

**Figure 22:**
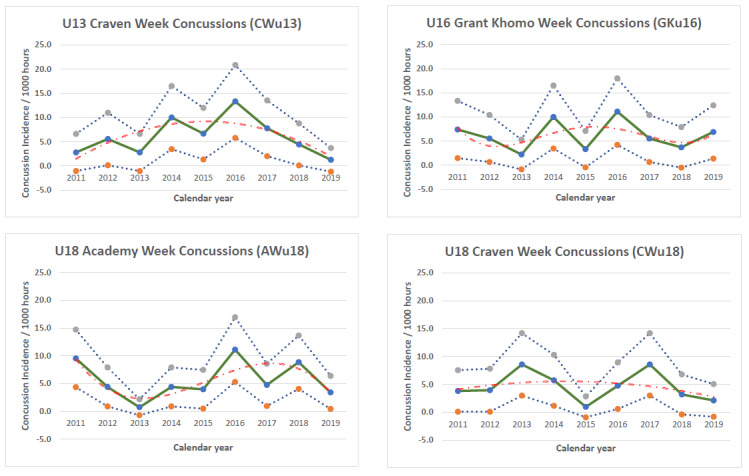
Concussion incidence and 95%CI for each SARU Youth Week tournament, from 2011 – 2019. The dashed orange line represents the polynomial trend

The changes in concussion incidence across individual tournaments fluctuated over time, increased initially from around 2013 with augmented concussion education, awareness and stricter protocols but have mostly decreased thereafter since about 2016 (Figure 22). The concussions at the CWu13, AWu18 and CWu18 decreased from 2018 to 2019. The CWu13 has continued to decrease since 2016, and the CWu18 since 2017. Only in the GKu16 did the concussions increase marginally from 2018 to 2019. This should be monitored in the future.

In Figure 22, the CWu13 and AWu18 trendlines account for 50% and 60% of the variance in concussion incidence per 1000 player hours (R^2^ = 0.50 and 0.60, respectively), confirming the trend of decreasing concussions. CWu18 concussions are also on a downward trend, however, the trendline can only account for 10% of the variance in injury incidence per 1000 player hours (R^2^ = 0.10). GKu16 concussions after moving downwards in 2016, are on a slight upward trajectory in 2019, but the trendline can only account for 20% of the variance in injury incidence per 1000 player hours (R^2^ = 0.20)).

## Figures and Tables

**Table 1:** Number and injury incidence (95% CI)/1000 player hours of Medical Attention and Time-Loss injuries in the 2019 SARU Youth Week tournaments.

	Medical Attention Injuries		Time-Loss Injuries	

	Number	Incidence	Number	Incidence
**CWu13**	47	59 (42 – 76)	4	5 (0 – 10)*
**GKu16**	64	74 (56 – 92)	23	26 (16 – 37)
**AWu18**	28	19 (12 – 26)*	25	17 (10 – 24)
**CWu18**	44	47 (33 – 60)	21	22 (13 – 32)

***Combined Total***	***183***	***45 (38 – 51)***	***73***	***18 (14 – 22)***

* Significantly different across tournaments

**Table 2:** Number of Medical Attention and Time-Loss injuries. Data expressed per match and per hour played in the 2019 SARU Youth Week tournaments.

Tournament	Number of matches	Match duration (mins)	Medical Attention injuries/match	Time-Loss injuries/match	Medical Attention injuries/hour	Time-Loss injuries/hour
**CWu13**	32	50	1.5	0.1*	1.8	0.2*
**GKu16**	29	60	2.2	0.8	2.2	0.8
**AWu18**	42	70	0.7*	0.6	0.6*	0.5
**CWu18**	27	70	1.6	0.8	1.4	0.7

***Combined Tournament Average***	***33***	***63***	***1.5***	***0.6***	***1.5***	***0.6***

* Significantly different across tournaments

**Table 3:** Injury incidence (95% CI)/1000 player hours of Time-Loss injuries to the Tackler and Ball Carrier roles, and the Ruck phase of play for the 2019 SARU Youth Week tournaments.

Tournament	Tackler	Ball Carrier	Ruck
**CWu13**	0	3 (0 – 6)	1 (0 – 4)
**GKu16**	6 (1 – 11)	9 (3 – 16)	7 (1 – 12)
**AWu18**	6 (2–10)	2 (0 – 4)	5 (2 – 9)
**CWu18**	7 (2 – 13)	7 (2 – 13)	3 (0 – 7)

***Combined total***	***5 (3 – 7)***	***5 (3 – 7)***	***4 (2 – 6)***

**Table 4:** Injury incidence (95% CI)/1000 player hours of Time-Loss injuries at the 2019 SARU Youth Week tournaments grouped as Joint/Ligament, Muscle/Tendon and Central Nervous System (CNS) injuries.

Tournament	Joint/Ligament	Muscle/Tendon	CNS
**CWu13**	4 (0 – 8)	0	1 (0 – 4)
**GKu16**	8 (2 – 14)	5 (0 – 9)	7 (1 – 12)
**AWu18**	10 (5 – 15)*	1 (0 – 2)*	3 (0 – 6)
**CWu18**	10 (3 – 16)	5 (1 – 10)	2 (0 – 5)

***Combined Total***	***8 (5 – 11)*** *	***2 (1 – 4)*** *	***3 (2 – 5)***

* Significantly different between Joint/Ligament and Muscle/Tendon injury types

**Table 5:** Proportion (%) and incidence (95% CI)/1000 player hours of Time-Loss injuries, grouped by body location, in the 2019 SARU Youth Week tournaments.

	Proportion of injuries (%)	Incidence (95% CI)/1000 player hours
**Head & Neck**	26	5 (3–7)
**Trunk**	6	1 (0–2) *
**Upper Body**	26	5 (3–7)
**Lower Body**	38	7 (4–9)

* Significantly lower than the other grouped Body Locations

**Table 6:** Injuries grouped according to the IOC recommended categories of Tissue and Pathology types for the 2019 SARU Youth Week tournaments.

Tissue	Injuries	Incidence	Mean time loss

*Pathology*	n	Injuries per 1000 hours (95% CI)	Days (95% CI)

**Muscle/Tendon**	10	2 (1 to 4)	39 (0 to 79)
* * *Muscle strain*	9	2 (1 to 4)	38 (0 to 84)
* * *Tendon injury*	1	0.2 (0 to 1)	22
**Nervous**	14	3 (2 to 5)	35 (4 to 65)
* * *Concussion*	14	3 (2 to 5)	35 (4 to 65)
**Bone**	11	3 (1 to 4)	59 (17 to 101)
* * *Fracture*	11	3 (1 to 4)	59 (17 to 101)
**Ligament/Joint Capsule**	33	8 (5 to 11)	38 (14 to 62)
* * *Ligament sprain*	21	5 (3 to 7)	32 (7 to 58)
* * *Joint injury*	12	3 (1 to 5)	54 (1 to 108)
**Superficial tissue/skin**	5	1 (1 to 2)	10 (0 to 21)
* * *Contusion (superficial)*	4	1 (0 to 2)	13 (0 to 26)
* * *Laceration*	1	0.2 (0 to 1)	1

***TOTAL***	**73**	**18 (14 – 22)**	**34 (20 to 48)**

Where n = 1, mean Time-Loss reflects the total Time-Loss days. Estimated severity for Time-Loss was used from data provided by the Tournament Doctors at the venue, when real-time severity was not able to be determined.

**Table 7:** Number of injuries and injury rates (95% CI)/1000 exposure hours of players who started the match and those who came on as substitutes in the 2019 SARU Youth Week tournaments.

	Started match		Substitution	

	Number	Incidence	Number	Incidence
**CWu13**	4	5 (0–10)*	0	0*
**GKu16**	21	24 (14–35)	2	2 (0–6)
**AWu18**	19	13 (7–19)	6	4 (1–7)
**CWu18**	20	21 (12–30)	1	1 (0–3)

***Combined Total***	***64***	***16 (12–19)***	***9***	***2 (1–4)***

* Significantly lower than GKu16 and CWu18 tournaments

**Table 8:** Number and incidence of concussions (95% CI)/1000 player hours at the 2019 SARU Youth Week tournaments.

Tournament	Number	Incidence	Number of matches per concussion event
**CWu13**	1	1 (0 – 4)	32
**GKu16**	6	7 (1 – 12)	5
**AWu18**	5	3 (0 – 6)	8
**CWu18**	2	2 (0 – 5)	14

***Combined Total***	***14***	***3 (2 – 5)***	***9***
